# Exploration-Based SLAM (e-SLAM) for the Indoor Mobile Robot Using Lidar

**DOI:** 10.3390/s22041689

**Published:** 2022-02-21

**Authors:** Hasan Ismail, Rohit Roy, Long-Jye Sheu, Wei-Hua Chieng, Li-Chuan Tang

**Affiliations:** 1 Department of Mechanical Engineering, National Yang Ming Chiao Tung University, Hsinchu 30010, Taiwan; hasan.ismail.ft@um.ac.id (H.I.); rohit.me08g@nctu.edu.tw (R.R.); newton4538.eo85g@nctu.edu.tw (L.-C.T.); 2 Department of Mechanical Engineering, Chung Hua University, Hsinchu 30012, Taiwan; ljsheu@chu.edu.tw

**Keywords:** LiDAR, SLAM, active localization, exploration

## Abstract

This paper attempts to uncover one possible method for the IMR (indoor mobile robot) to perform indoor exploration associated with SLAM (simultaneous localization and mapping) using LiDAR. Specifically, the IMR is required to construct a map when it has landed on an unexplored floor of a building. We had implemented the e-SLAM (exploration-based SLAM) using the coordinate transformation and the navigation prediction techniques to achieve that purpose in the engineering school building which consists of many 100-m^2^ labs, corridors, elevator waiting space and the lobby. We first derive the LiDAR mesh for the orthogonal walls and filter out the static furniture and dynamic humans in the same space as the IMR. Then, we define the LiDAR pose frame including the translation and rotation from the orthogonal walls. According to the MSC (most significant corner) obtained from the intersection of the orthogonal walls, we calculate the displacement of the IMR. The orientation of the IMR is calculated from the alignment of orthogonal walls in the consecutive LiDAR pose frames, which is also assisted by the LQE (linear quadratic estimation) method. All the computation can be done in a single processor machine in real-time. The e-SLAM technique leads to a potential for the in-house service robot to start operation without having pre-scan LiDAR maps, which can save the installation time of the service robot. In this study, we use only the LiDAR and compared our result with the IMU to verify the consistency between the two navigation sensors in the experiments. The scenario of the experiment consists of rooms, corridors, elevators, and the lobby, which is common to most office buildings.

## 1. Introduction

Navigation is the basic function of autonomous vehicles in daily and industrial applications. For outdoor environment, vehicle localization and navigation can be achieved with the assistance of global positioning systems (GPS). However, in the indoor environment, the traditional navigation technology will fail due to the lack of satellite positioning signals [1]. Vehicle cruising in the indoor space can be localized and navigated through some characteristic graphics or marked points if there is a premade map of the space. If it is moving in an unknown environment, the localization and mapping must be carried out simultaneously. However, accurate localization requires an accurate map and vice versa. The localization and mapping must be concurrently performed which creates a complex problem called simultaneous localization and mapping (SLAM) first proposed by Hugh Durrant-Whyte and John J. Leonard [2,3,4].

Sensors are the way robots or self-driving vehicles perceive their environments. The selection and installation of sensors determine the specific form of observation results, and also affect the difficulty of SLAM problems. According to the major sensor, simultaneous localization and mapping can be divided into Visual-SLAM and LiDAR-SLAM.

Visual SLAM uses the vision camera as the major sensor to find features in the image and then use these features to match the known image for localization. Inspired by human vision, some studies use stereo cameras to perform SLAM [5,6,7,8]. In addition, monocular cameras can construct SLAM [9,10], including feature description methods [11,12], LSD-SLAM [13] and SVO [14]. An effective Visual SLAM needs to extract and match features, which requires huge amounts of calculations and is time-consuming for modern computers. The relationship between the image coordinates of the matching points and the space coordinates is nonlinear. Matching these features will introduce many constraints that make the process of the optimization problem of multiple variables more complicated and slower. These two difficulties make the Visual SLAM system still a very challenging task. In addition, the vision camera cannot extract features from textureless regions, which also limits the use of Visual SLAM. LiDAR SLAM mainly uses LiDAR for sensing environments. The relative movement and pose change of the laser radar can be determined by matching and comparison of the point clouds at different times. Its advantages are stability, simple, accurate map information and less calculation than Visual SLAM. It has been widely used, most of which use iterative closest point (ICP) [15,16,17,18] scan-matching technique, including Scan to Scan [19,20,21,22,23] and Scan to Map [24,25,26,27]. For the comparison of various LiDAR-based SLAMs, please refer to the literature [28].

The most important core of solving the problem of SLAM is finding the motion and observation models to provide efficient and consistent calculations for estimation and measurement updates. However, due to the presence of noise in the measurement, the subsequent estimation will transmit the noise error. The calculation algorithm of SLAM can be roughly divided into two categories: filters and optimization methods. The filter methods are based on two kinds: Gaussian filter (GF) and particle filter (PF). The Gaussian filters including Kalman filter (KF) and extended Kalman filter (EKF) separately deal with linear and nonlinear issues. The optimization method commonly uses pose graph optimization to minimize the square errors to solve the SLAM. In addition, since artificial intelligence (AI) has prevailed in recent years, learning methods are also used to solve the SLAM problem [29,30,31,32].

The principle of the Kalman filter is to use the current state information and input to infer the output at the next moment. Assuming that the noise follows a Gaussian distribution, the recursive algorithm is used to effectively estimate the pose of the vehicle. If the error model is a Gaussian distribution, the accuracy of the estimated result will be quite good. The advantage of the Kaman filter lies in its fast convergence. The disadvantage is that the error model must be a Gaussian distribution as otherwise the estimated result will lack accuracy. Besides, the size of the state vectors directly affects the dimension and time of the matrix operation. The SLAM based on the Kalman filter assumes that the system is linear, but in fact, the problem of SLAM is rarely linear. Therefore, the Extended Kalman filter (EKF) was developed to solve the nonlinear positioning problem. The EKF-SLAM was first proposed by Smith et al. [33], after which many researchers further improved this method [34,35,36].

The particle filter SLAM [24,37,38] is a recursive filter using a Monte Carlo algorithm to estimate the pose and movement path of the vehicle. Random distribution of particles is sampled. Each particle in the sample represents the vehicle trajectory and a map. These particles are weighted to fit a closer actual environment, and re-sampling is performed according to the distribution result. As the robot continues to move in space, particles will gradually gather at the most likely pose of the robot in space, and the map will be updated simultaneously. Although the particle filter algorithm can be used as an effective method to solve the SLAM problem, the main problem is that a large number of samples are required to approximate the posterior probability density of the system. The more complex environment the robot faces, the more samples are needed to describe the posterior probability and the higher the complexity of the algorithm.

In addition to solving SLAM problems with filters, graph-based SLAM [39,40,41] takes a different approach for estimating a robot trajectory. It creates a pose graph, whose node corresponds to a pose of the robot during mapping and every edge between two nodes corresponds to a spatial constraint between them. The bundle adjustment is performed to estimate trajectory and map from the input feature tracks. Cartographer is a Google open-source project developed in 2016. It is a system that provides 2D and 3D real-time SLAM under multiple platforms and sensor configurations. The main theory of Cartographer is to eliminate the cumulative error during mapping through loop closure detection.

Regarding the map construction of SLAM, there are three commonly used maps [42]: Occupation grid, Feature-based map and Topological map.

Grid map: The most common way for robots to describe the environment map is a grid map or an occupancy map. A grid map divides the environment into a series of grids, where a value is given in each grid to represent the probability of being occupied [43,44].

Feature map: It relates geometric features (such as points, lines, and surfaces) to represent the environment, which is commonly used in Visual SLAM technology [45,46,47,48,49].

Topological map: It is a relatively more abstract map form. It expresses the indoor environment as a topological structure diagram with nodes and related connecting lines. The nodes represent important locations in the environment (corners, doors, elevators, stairs, etc.). Edge represents the connection relationship between nodes, such as corridors. This method only records the topological link relationship of the environment. For example, when a sweeping robot wants to clean a room, such a topological map will be established [50,51,52,53,54,55].

Regardless of Visual SLAM or LiDAR SLAM, most systems need auxiliary data for vision or LiDAR through other sensors such as inertial measurement unit (IMU) or odometer, otherwise the SLAM system will be difficult to operate. The multi-sensor fusion SLAM increases the costs of both equipment and calculation. The operation of this kind of system is also a considerable burden. This study proposes a set of algorithms that uses only LiDAR signals to perform SLAM. The algorithm can quickly find mutually perpendicular walls in space to be referenced through simple geometric rotation features. It is unnecessary to compare the original building layout to complete the localization and map construction of rooms, floors and buildings. The equipment is relatively simple and the amount of required calculation is greatly reduced compared with previous methods. It can be used as a real-time SLAM in the unexplored building.

## 2. LiDAR Point Cloud

LiDAR originated in the early 1960s, shortly after the invention of the laser, by emitting and receiving laser beams, LiDAR uses the time-of-flight (ToF) method to obtain an object’s position and its characteristics. This method is almost independent of illumination conditions, has a long detection time range and high accuracy. Its first application came from meteorology, where the National Center for Atmospheric Research used it to measure clouds and pollution [56]. There is a growing interest in portable and affordable three-dimensional LiDAR systems for new applications [57,58,59,60,61]. Many examples of rotating single-beam LiDAR can be found in the literature [62,63,64,65]. The addition of a degree-of-freedom to build a rotating multi-beam LiDAR has the potential to become a common solution for affordable rapid full-3D high-resolution scans [66,67,68,69,70].

**Figure 1:**
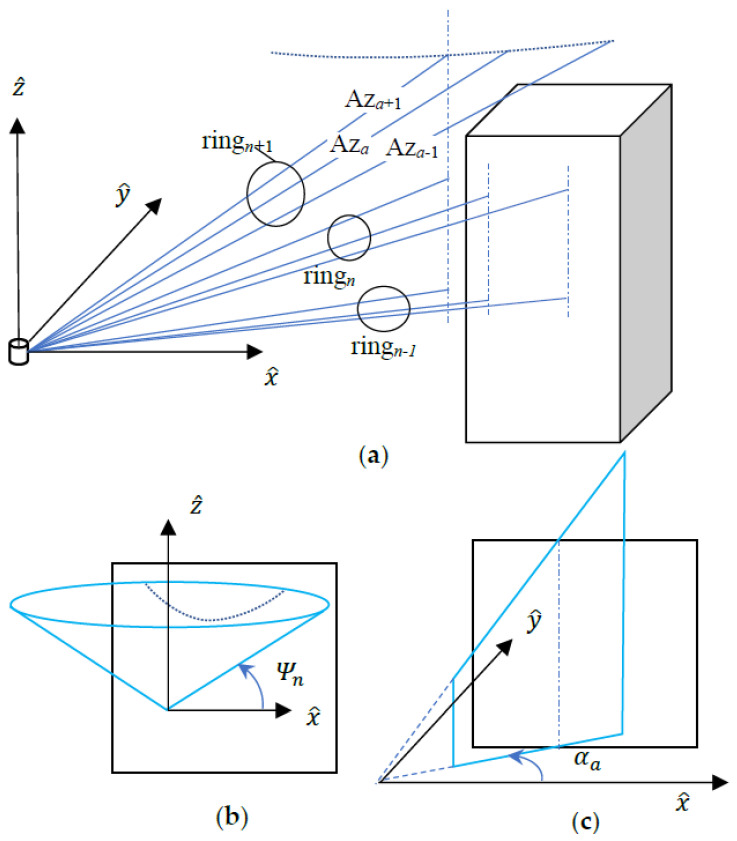
LiDAR data points: (**a**) on different walls, (**b**) ring projection, and (**c**) azimuth projection.

The LiDAR scanner rotates at a constant speed about the rotary axis, which is defined per convention as the $\hat{Z}$ axis, and sends an array of rays. The number of rays is denoted as the number of rings, which are shot from the LiDAR center outward into the space in different angles denoted as the ring angle Ψ. The constant speed of rotation of the linear array results in the same laser ray to form a ring in the space and the speed of rotation that multiply the sampling time of sensing is known as the azimuth angle of resolution. It can be observed from Figure 1a that the LiDAR ray points are projected on the walls according to its rays in different ring and azimuth angles. The rays with the same ring angle $\Psi_{n}$ but different azimuth angles form a cone surface as shown in Figure 1b. A nonlinear curve can be determined when the cone surface intersects a flat wall. The rays with the same azimuth angle $\alpha_{a}$ but different ring angles form a planar surface as shown in Figure 1c. A vertical line can be detected when the planar surface intersects a flat wall.

### 2.1. LiDAR Point Cloud and Mapping via Transformation and Inverse Transformation

We first define the elevation angle of the *n*-th ring as $\Psi_{n}$, the swing angle of the *a*-th ray on the same LiDAR image ring as $\alpha_{a}$, and the individual ray distance is detected from the returned signal through time-of-flight conversion into distance ${\hat{r}}_{n.a}$ in meter. The LiDAR data point can be presented in a three-dimensional tensor as $\Lambda_{n.a}=\Lambda \left({\hat{r}}_{n.a},\Psi_{n},\alpha_{a}\right)$. The spherical transformation matrix from the spherical coordinate to the rectangular coordinate system can be expressed as follows.

(1) $$R\left(\Psi_{n},\;\alpha_{a}\right)=\left[\begin{array}{ccc} cos\alpha_{a}cos\Psi_{n} & cos\alpha_{a}sin\Psi_{n} & sin\alpha_{a}\\ sin\alpha_{a}cos\Psi_{n} & sin\alpha_{a}sin\Psi_{n} & -cos\alpha_{a}\\ sin\Psi_{n} & -cos\Psi_{n} & 0 \end{array}\right]$$

The LiDAR point ${\hat{P}}_{n,a}$ can then be presented in the rectangular coordinate system as follows.

(2) $${\hat{P}}_{n,a}=\left[\begin{array}{c} {\hat{p}}_{x}\\ {\hat{p}}_{y}\\ {\hat{p}}_{z} \end{array}\right]=\Gamma \left\{\Lambda_{n.a}\right\}=R\left(\Psi_{n},\;\alpha_{a}\right)\left[\begin{array}{c} {\hat{r}}_{n.a}\\ 0\\ 0 \end{array}\right]={\hat{r}}_{n.a}\left[\begin{array}{c} cos\alpha_{a}cos\Psi_{n}\\ sin\alpha_{a}cos\Psi_{n}\\ sin\Psi_{n} \end{array}\right]$$

Equation (2) is considered as the transformation from $\Lambda_{n.a}$ to ${\hat{P}}_{n,a}$. The inversion of a point in the Cartesian coordinate system to the three-dimensional LiDAR data tensor is given as follows.

(3) $$\begin{array}{c} \Psi =tan^{-1}\frac{{\hat{p}}_{y}}{{\hat{p}}_{x}}\\ \alpha =tan^{-1}\frac{{\hat{p}}_{z}}{\sqrt{{\hat{p}}_{x}{}^{2}+{\hat{p}}_{y}{}^{2}}}\\ \hat{r}=\sqrt{{\hat{p}}_{x}{}^{2}+{\hat{p}}_{y}{}^{2}+{\hat{p}}_{z}{}^{2}}\;\;\;\; \end{array}$$

The procedure with discretizing the analog value of Ψ and α into the discrete value of $\Psi_{n}$ and $\alpha_{a}$ according to the LiDAR resolution is defined as the inverse transformation of Γ.

(4) $$\Lambda \equiv \Lambda_{n.a}=\Gamma^{-1}\left\{\hat{P}\right\}$$

Following the convention of coordinate frame transformation used in robotics, the location and orientation in the Cartesian coordinate system is defined as the LiDAR pose frame which is given as follows.

(5) Lk=[LkxLkyLkγLkp]

Lkx denote the abosolute x translation, Lky denote the absolute y translation, Lkγ is the abosolute LiDAR azimuth angle, and Lkp is the abosolute LiDAR pitch angle in the view of global coordinate system. Converting the cloud point Λ11 from its LiDAR pose frame L1 to the LiDAR pose frame L0 denoted as Λ10, we will have to go through the procedure as follows.

(6) P^1=Γ{Λ11(r^n.a,Ψn,αa)}

P^1 is the Cartesian coordinate in pose frame L1.

(7) Λ10=Γ−1{Θ10P^1}

Λ10 is the LiDAR data point obtained in pose frame L1 and converted into the LiDAR data point in pose frame L0. Θ10 is a homogenous transformation from pose frame L1 to the pose frame L0, which will be described in the following sections. The consecutive LiDAR motion may also be updated in a sequence of transformations from LiDAR pose Lk and mapped back to the coordinate frame defined from the LiDAR pose frame L0.

(8) Λk0=Γ−1{Θ10·Θ21·…·Θkk−1P^k}

After the LiDAR transformation Γ and its inverse transformation $\Gamma^{-1}$ are adequately defined for a particular LiDAR such as a LiDAR with 32 rings and one degree azimuth resolution, one is able to move the LiDAR from one position to the other position to consecutively gather more points into the cloud of the frame. This procedure is known as mapping. The point cloud mapping shall be much clearer to the 3D scene of a space such as a room of a lobby. The conventional way to do the mapping is based on data fusion from different sensors, such as the camera, the IMU, GPS, and the accelerometer. The data fusion is costly in either processing time or money in equipment as well as their installation, calibration and maintenance. This paper proposes a method which strictly utilizes the LiDAR sensor to achieve mapping provided that the lattice structured walls can be seen all the time through the vision of the LiDAR.

**Figure 2:**
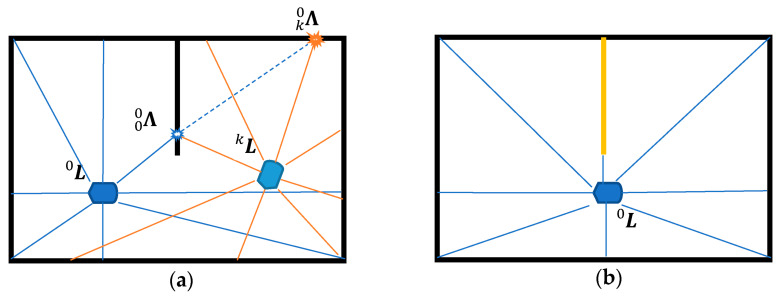
Example of (**a**) mapping conflict of a partitioned room, (**b**) unobservable wall.

After a series of point cloud conversions, we can obtain multiple points with different ${\hat{r}}_{n.a}$’s but same $\Psi_{n}$, $\alpha_{a}$. The procedure to gather LiDAR data points is the first step of mapping. It may result that a mapping conflicts with one another from a different view point of space. Figure 2a depicts the mapping conflict when the LiDAR is moving from L0 to Lk. From the view of L0, the ray distance of Λ00 is shorter than when it is mapped from Lk which is Λk0. In this case, we will have to change the room index and store the adjacency information of the neighboring rooms. In case of a partitioned room, we may need multiple buffers of point cloud data to describe the same room which will be introduced in the following sections. Figure 2b depicts the wall that is aligned with one of the ray directions that is unobservable by the LiDAR scan. It is then necessary to properly select the reference frame L0 that can represent all important features clearly.

### 2.2. LiDAR Mesh and Filtering

**Figure 3:**
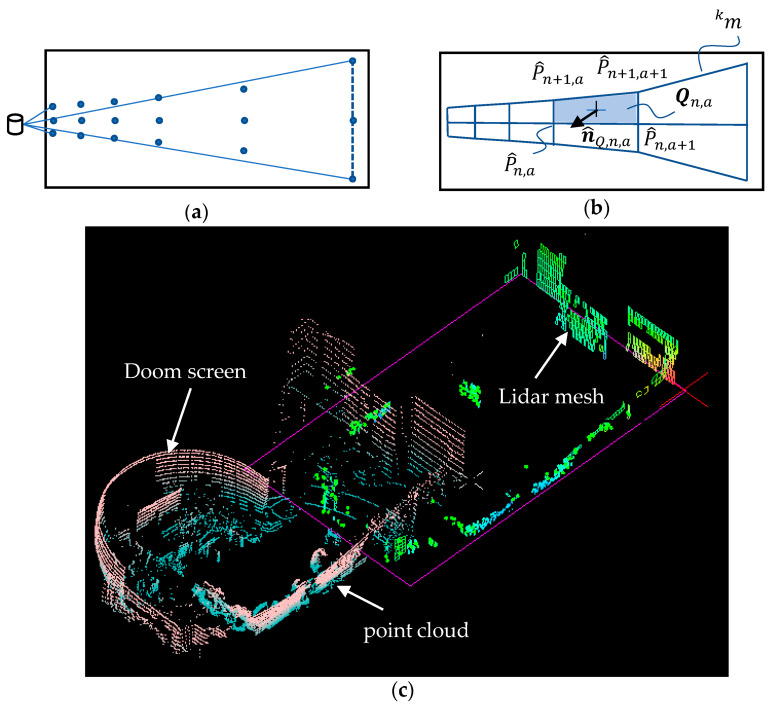
LiDAR mesh formation: (**a**) point cloud, (**b**) polygonal quads, and (**c**) result.

The LiDAR points form a set of grid points different from the rectangular grids on the wall even when the LiDAR is accurately positioned upright as shown in Figure 3a. The grid points are in a bell shape of which the height is smaller where the LiDAR is closer to the wall. The grid points may be converted into polylines horizontally for each ring and vertically for each azimuth angle. The horizontal and vertical line segments form a mesh m(kT)≡mk at the *k*-th sampling time T period as shown in Figure 3b. The mesh is composed of different quads, each of the individual quads has its four vertices and a normal vector ${\hat{n}}_{Q,n,a}$ of the quad $Q_{n,a}$. It may be determined from its four vertices.

(9) $${\hat{n}}_{Q,n,a}=\frac{\left({\hat{P}}_{n,a+1}-{\hat{P}}_{n,a}\right)\times \left({\hat{P}}_{n+1,a}-{\hat{P}}_{n,a}\right)}{\left|\left({\hat{P}}_{n,a+1}-{\hat{P}}_{n,a}\right)\times \left({\hat{P}}_{n+1,a}-{\hat{P}}_{n,a}\right)\right|}$$

The mesh mk may be filtered through three proposed conditions as follows.

(a) The cluster condition: the far away points resulted from the mirror reflection or through the windows shall be removed by the filter;
(b) The vertical condition: the quads $Q_{n,a}’s$ whose ${\hat{n}}_{Q,n,a}$ composes a large z component formed from the discontinuity of the surfaces. For example, the adjacent rays which one shoots to a wall and then shoots to a cabinet surface individually, shall also be removed;
(c) The aspect ratio condition: the aspect ratio of the quad $Q_{n,a}$, which are distorted too much due to the mirror reflection or complicated shape of the facilities existing in the offices, shall be filtered out.

The remaining LiDAR mesh mk as shown in Figure 3b may be discontinuous in either direction of X^k, Y^k, and Z^k. The discontinuities can be used later for segmentation of different walls composing the boundary of the rooms.

After the processing of the proposed conditions, the LiDAR mesh will keep only those with grid patterns. The evidence for this is depicted in Figure 3c as an example. The doom screen in the room is filtered out from the proposed conditions, which is not grid-like. The proposed method is effective only for the vertical walls, doors or cabinets which have the grid pattern. It is common that the walls are used to partition spaces in typical office buildings.

### 2.3. Mesh Projection and Initial Axis Finding

**Figure 4:**
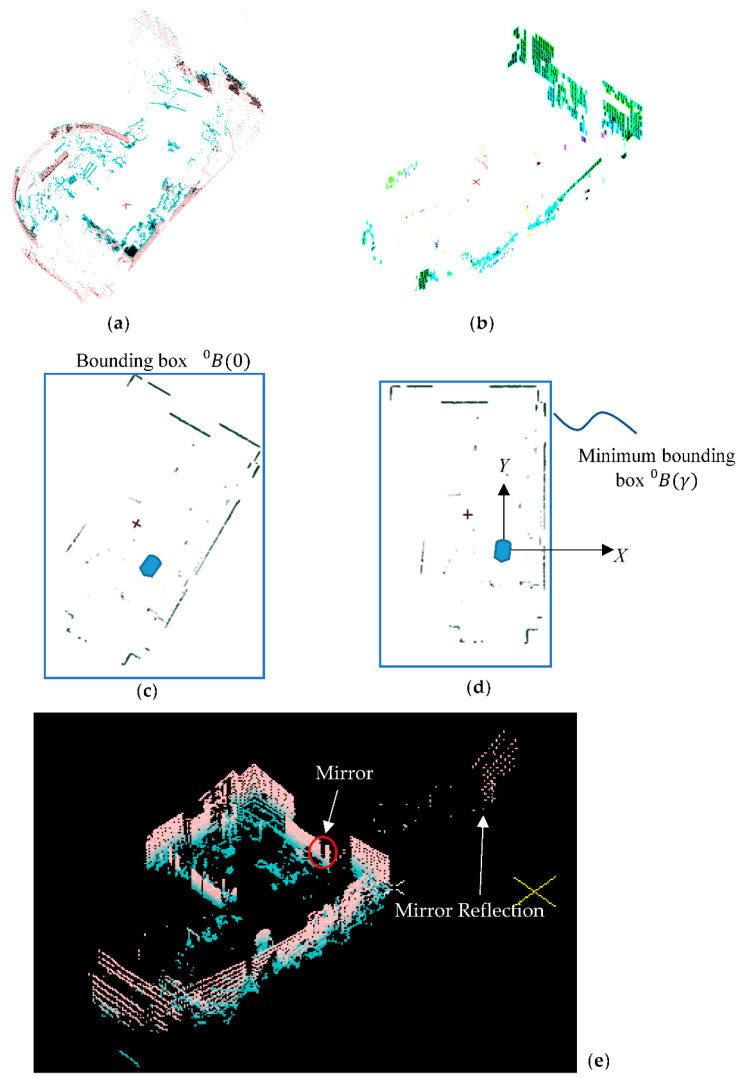
Initial axis finding procedure: (**a**) point cloud, (**b**) mesh filtering, (**c**) *XY* projection, (**d**) minimum area bounding box rotation, (**e**) the aliasing data from the mirror.

After the filtering, we can determine a bounding box B(kT)≡Bk for the mesh mk, which is formed from the envelope of the cloud points ${\hat{P}}_{n,a}$ collected during the sampling time *T*. It is also assumed that the LiDAR is positioned rather vertical to the ground and it is in a square room when k=0. The bounding box B0 is then projected on the $\hat{X}\hat{Y}$ plane. The LiDAR image projection is rotated on the $\hat{X}\hat{Y}$ plane with an angle γ about the $\hat{Z}$ axis to find the minimum area of B0(γ) under the rotation of γ. At the minimum area rotation, we determine the principal axis as the ***X*** and ***Y*** axes of this room and, if necessary, the global coordinate system as shown in Figure 4d. 

After the above procedures, the normal vector ${\hat{n}}_{Q,n,a}$ of the quad $Q_{n,a}$ is used to calibrate the pitch angle of the LiDAR which can be shaken by the vehicle vibration. We first identify the individual quads $Q_{n,a}’s$ which fulfills the following conditions and categorize them into *X* walls and *Y* walls.

(10) $${\hat{n}}_{Q,n,a}\cdot \left[\begin{array}{c} 0\\ \pm 1\\ 0 \end{array}\right]\geq 1-\delta \;\mathrm{for}\;\mp \hat{X}\;\mathrm{wall}$$

(11) $${\hat{n}}_{Q,n,a}\cdot \left[\begin{array}{c} \pm 1\\ 0\\ 0 \end{array}\right]\geq 1-\delta \;\mathrm{for}\;\mp \hat{Y}\;\mathrm{wall}$$

δ is a small value, which is recommended to be 0.02. Taking the average of the *X* wall normal vectors to obtain a unit vector ${\hat{n}}_{Xwall}$ and Y wall normal vectors to obtain a unit vector ${\hat{n}}_{Ywall}$ and taking cross product on the two average normal vectors, we are able to determine the floor normal vector as follows.

(12) $${\hat{n}}_{floor}={\hat{n}}_{Xwall}\times {\hat{n}}_{Ywall}$$

The rotational transformation of the LiDAR point from the LiDAR Coordinate System $\hat{X}\hat{Y}\hat{Z}$ to the global coordinate system can be achieved by the following operators.

(13) $$P_{n,a}=\left[\begin{array}{c} p_{x}\\ p_{y}\\ p_{z} \end{array}\right]=R{\hat{P}}_{n,a}$$

R denotes the rotational transformation as follows.

(14) $$R=\hat{X}\left(\alpha \right)\hat{Y}\left(\beta \right)\hat{Z}\left(\gamma \right)$$

The angles α and s with respect to the *X* and *Y* walls respectively are given in the following.

(15) $$\begin{array}{c} \alpha =-sin^{-1}\left({\hat{n}}_{floor}\cdot \left[\begin{array}{c} 0\\ 1\\ 0 \end{array}\right]\right)\\ \beta =-sin^{-1}\left({\hat{n}}_{floor}\cdot \left[\begin{array}{c} 1\\ 0\\ 0 \end{array}\right]\right) \end{array}$$

After the above transformation, we can align the initial LiDAR image and identify the axes of the global coordinate system. The minimum area method as the search for the rotation angle γ about the $\hat{Z}$ axis is valid in the initial stage of the e-SLAM. The following conditions are preferred to establish the first LiDAR post frame L0 with good accuracy.

(1) Rectangular room condition: the room where the IMR starts must be a rectangular room with orthogonal walls to allow the minimum area method to find the principle axes properly;
(2) No mirror condition: the room must initially be with no mirror reflecting the LiDAR ray. Without this condition, the aliasing LiDAR data can misinterpret the rectangular room.

**Figure 5:**
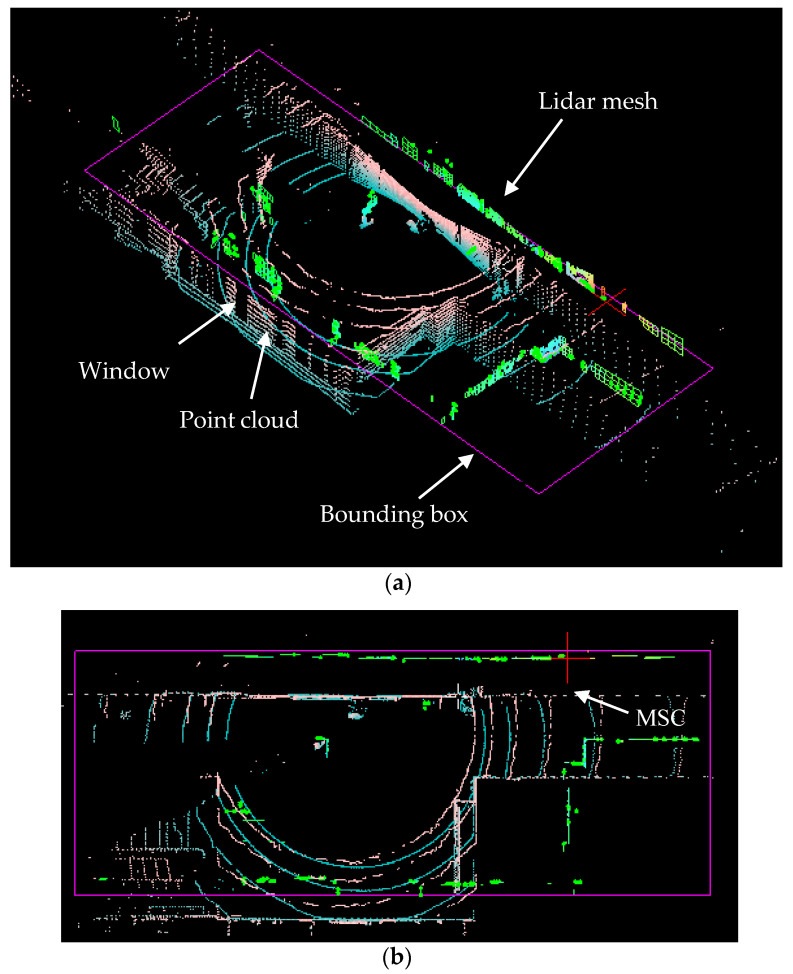
Initial axis finding with minimum bounding box: (**a**) perspective view and (**b**) top view.

These conditions may be unavailable in a situation where the IMR is started from the center of the lobby or an open space as shown in Figure 5a. However, in many situations, there are good long reference walls, the minimum area method will bring the bounding box to align with the longest wall as shown in Figure 5b. On the other hand, if there is a mirror in the initial position, then the aliasing LiDAR data that expand the room can mislead the minimum area method to an incorrect rotation as shown in Figure 4e. Drawbacks of the minimum area method include ±90° rotation error, the fluctuation of the rotation due to the unclear wall information, and the large angular error due to the envelope of the LiDAR image which is not rectangular in the $\hat{X}\hat{Y}$ plane projection.

### 2.4. Wall Corner as Land Mark

**Figure 6:**
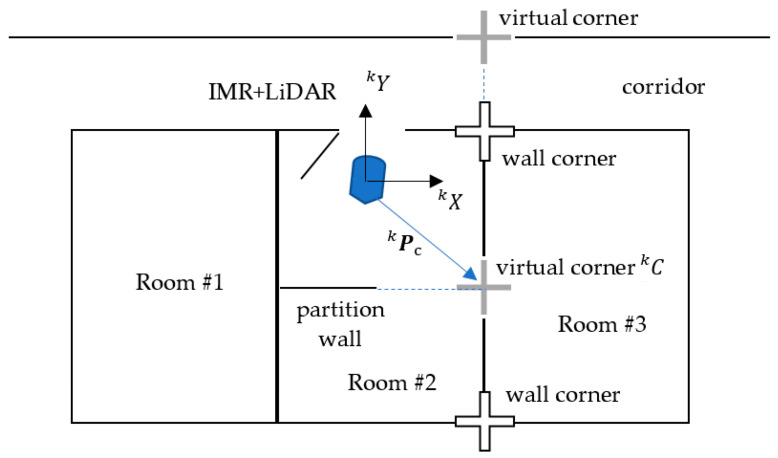
Wall corners, virtual corners and the most significant corner (MSC) Ck.

The intersection of X and Y walls are the wall corner, which can be either an existing or a virtual corner. For example, there could be a wall on the corridor referred to as the *X* wall as viewed from the room while the other side of the corridor walls are referred to as the *Y* walls. There are not any actual corners, hence, called the virtual corner in the corridor as shown in Figure 6. The wall corners or virtual corners can be utilized as the land mark of the translation calculation, that is, how far the IMR (indoor mobile robot) is moving away from or approaching a known location landmark. The walls are extracted from the quads which are marked as the *X* walls and *Y* walls.

(16) Wx(kT)≡Wkx={Qn,a|nQ,n,a·[0±10]≥1−δ}Wy(kT)≡Wky={Qn,a|nQ,n,a·[±100]≥1−δ}

The importance of the wall is given with a weight proportional to the ray distance $\hat{r}$ which cannot be affected by the rotation pivoted at the LiDAR location. Hence, the histogram on *X* and *Y* directions may be written as follows.

(17) Hkx(y)=∑Q⊂Wkx,pky=yr^n,aHky(x)=∑Q⊂Wky, pkx=xr^n,a

The histograms have their stationary values defined as follows.

(18) ∂∂yHkx(ykc,i)=0∂∂xHky(xkc,i)=0

These stationary values can be sorted to obtain a set of wall corners or virtual corners as follows.

(19) Pkc,i≡[xkc,iykc,i]

The displacement xkc,i and ykc,i satisfy the following conditions in Lk pose frame.

(20) ∂∂yHkx(ykc,i)=0, ∂∂xHky(xkc,i)=0, i=1, 2, 3…

In the set of corners, Pkc≡Pkc,1 is defined as the location of the most significant corner (MSC) Ck discovered at *k*-th sampling time. Pkc,1 satisfies the following minimax condition.

(21) min(Hkx(ykc,1),Hky(xc,1))≥min(Hkx(ykc,i),Hky(xkc,i)), i=2, 3…

Note that Ck is a feature corner which is only an abstraction that the feature identification shall be achieved either in the histogram correlation or the LiDAR image comparison.

### 2.5. LiDAR with IMR Navigation

In the initial stage, i.e., k=0, the position of the most significant corner P0c  can be used to define the pose frame L0 when γ0 is derived from the minimum bouning box B0(γ0). The pitch angle is assumed eliminated during the mesh conversion process stated in Section 2.3 “Mesh Projection”, which is set to zero. Hence, the LiDAR pose frame is reduced into a three-dimensional tensor as follows.

(22) L0=[−P0cγ00]4×1→[−P0cγ0]3×1

The initial pose frame indicates that the origin of the pose frame is located at x0c,1 and y0c,1 offset with respect to the coordinate system when applying the rotation of γ0 angle about Z^0. The displacement and azimuth rotation of the LiDAR pose frame L(kT)≡ Lk during the later IMR navigation are calculated according to the initial pose frame L0.

(23) Lk=[Lktγk]3×1

Lkt denotes the translation of LiDAR.

(24) Lkt=−Pkc

**Figure 7:**
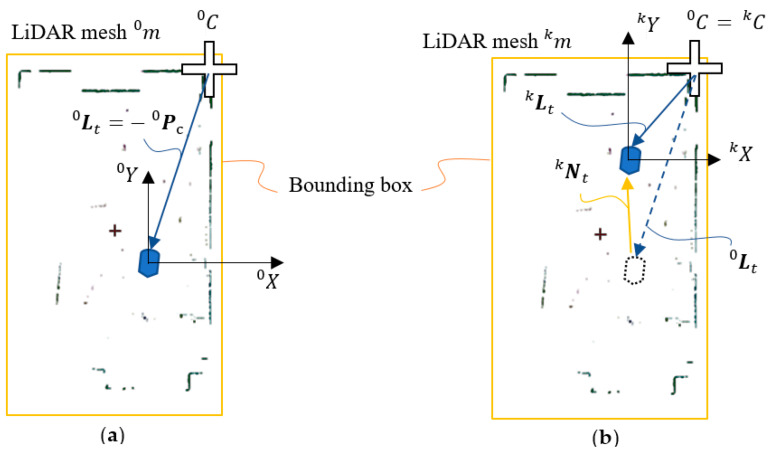
Determination of the (**a**) LiDAR pose frames, (**b**) IMR navigation.

The initial position of the IMR or LiDAR is located at −P0c, which is the origin of the pose frame L0. The initial orientation of LiDAR may be derived from the zero-azimuth angle direction relative to the X axis. In case that the most significant corner remained in the *k*-th sampling time of the LiDAR pose frame as shown in Figure 7, the translation Nkt and the rotation angle Nkγ of the IMR or LiDAR navigation can be written as follows.

(25) Lk=[NktNkγ]3×1=[Lkt−L0tγk−γ0]

It is convenient to attach the LiDAR pose frame to the actual pose of the IMR in the *k*-th sampling time. The translation and rotation can be calculated through the coordinate transformation such as the conventional robotics. When the LiDAR transformation Γ and its inverse transformation $\Gamma^{-1}$ are available, one is able to be move the LiDAR from one position to the other position to gather more and more points into the cloud frame. The mapping is achieved by converting the LiDAR point cloud from one scene to the other. The mapping becomes practical when the following two assumptions are fulfilled.

(1) The continuity of the rotation between LiDAR pose frames;
(2) The consistency of the most significant corner tracking is maintained even when the most significant corner changes through time.

To ensure both assumptions, the consistency of point cloud information has to be used in the verification. However, variations of the environment, such as objects moving, human coming into or leaving the scene, can create uncertainties to the consistency comparison. We then need a stochastic process to help us estimate the pose from the previous time to the consecutive time. Following the stochastic estimation, the rotation and translation finding procedures are discussed in the following sections.

### 2.6. Linear Quadratic Estimation for IMR Pose Prediction

The Linear Quadratic Estimation (LQE) may be applied to predict the LiDAR pose L˜k and its derivative L˙˜k through the following update scheme.

(26) X˜k6×1≡[L˜kL˙˜k]=A·(I−Kk·H)·X˜k−1+Kk·H·[Lk−1L˙k−1]

Lk−1 is the actual pose computed through the rotation and translation computation, which will be introduced in later sections. The actual velocity L˙k−1 may be updated using backward difference method and can be set to zero when it is not observed by the observation matrix H.

Kk is the Kalman gain at the *k*-th sampling, which is updated as follows.

(27) Kk6×3=Pk−1k·HT·(H·Pk−1k·HT+R)−1

Pk−1k is the prior error covariance matrix at the *k*-th sampling, which is given as follows.

(28) Pk−1k=A·Pk−1·AT+Q.

Pk is the posterior error covariance matrix at the *k*-th sampling, which is updated from the prior error covariance matrix as follows.

(29) Pk6×6=(I6×6−Kk·H)·Pk−1k

The transition matrix A  can be derived from Newton’s law of motion as follows.

(30) $$A_{6\times 6}=\left[\begin{array}{cc} I_{3\times 3} & T\cdot I_{3\times 3}\\ 0_{3\times 3} & I_{3\times 3} \end{array}\right]$$

The observation matrix H may observe only the pose and not the velocity of IMR as follows.

(31) $$H_{3\times 6}=\left[\begin{array}{cc} I_{3\times 3} & 0_{3\times 3} \end{array}\right]$$

The noise covariance matrices $R_{6\times 6}$ and $Q_{6\times 6}$ are related to the variance of the position and velocity individually. LQE yields the prediction of the position as well as the rotation of the IMR which carries the LiDAR. The most important feature is that LQE can yield a filtered result of L˙˜k prediction which leads to a range for the rotation and translation search of the LiDAR mesh mk.

### 2.7. Rotation Update Scheme Based on LQE

As stated previously, the minimum bounding box area method can be used in finding the rotation only during the initial stage. For the remaining steps k>0, we need to find the rotation γk based on the previous rotation angle γk−1 which is the previous LiDAR orientation. γk can only be in the range of α0L˙˜kγT±α1 in the vicinity of γk−1, where $\alpha_{0}$ is a factor of allowable variation and $\alpha_{1}$ is the allowable rotation change when the previous rotation speed is zero. We can apply a rotation angle γk to rotate the mesh points as follows.

(32) Pn,a=Z^(γk)P^n,a|γk−γk−1−α0L˙˜kγT|≤α1

**Figure 8:**
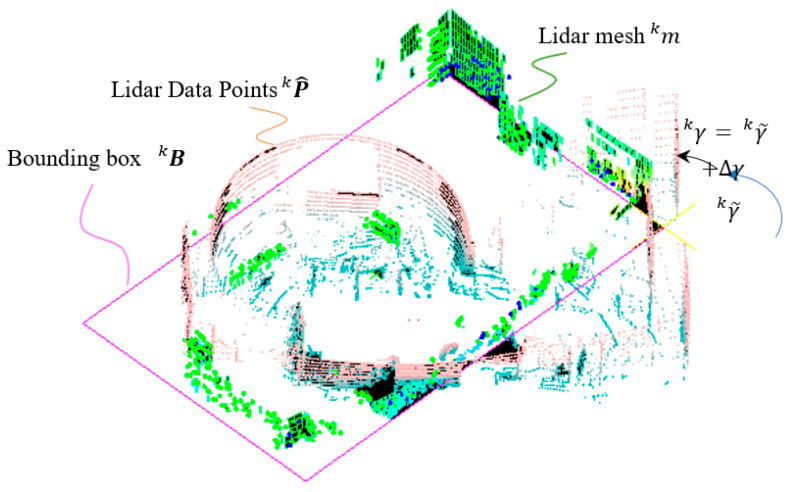
Determination of LiDAR rotation.

α0L˙˜kγT is the estimated rotation from the Kalman filter denoted as Δγ in Figure 8. The purpose of the rotation test is to find the minimum rotation difference between γk and γk−1 allowing the histogram values of the walls, i.e., Hkx and Hky, to be maximized. These can be done from one of the three methods stated as follows.

(1) Minimum bounding box area method: the same method for the rotation finding as in the initial stage, which may be working for the early time when k is small;
(2) Maximum total histogram value: maximize ∑Hkx+∑Hky;
(3) Maximum total number of quads on the bounding box; maximize the members in the set {Qn,a|Qn,a⊂Bk−1}.

The practical way is to use all three methods simultaneously and verify which one satisfies Equation (29). If all three methods fail, then find the γk which minimize |γk−γ˜k|. The incremental rotation can bring the mesh to align with the previous LiDAR mesh mk−1. The remaining difference between mk and mk−1 is the coincidence of the wall corners between the meshes.

### 2.8. Translation Update Scheme Based on Most Significant Corner (MSC) Transfer

**Figure 9:**
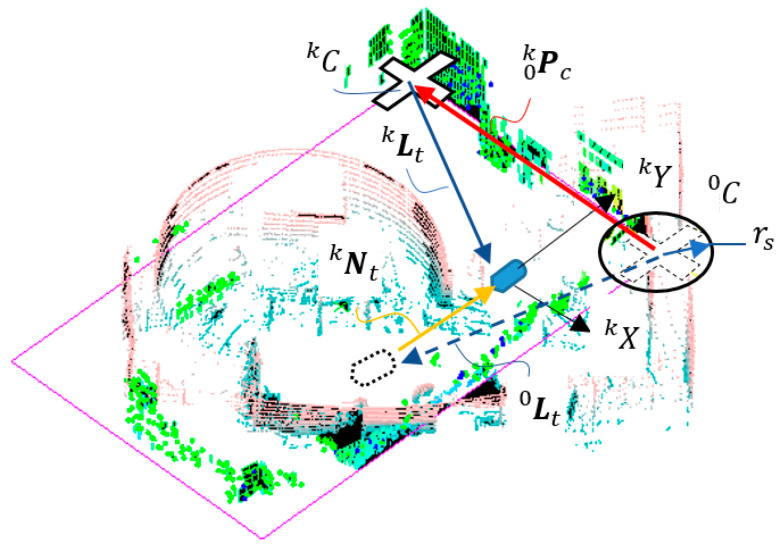
Determination of the previous MSC.

In case that the C0k  is not any more the most significant corner in the *k*-th sampling as shown in Figure 9, we will have to update the IMR navigation in the following way.

(33) Nk=[NktNkγ]3×1=[P0kc+(Lkt−L0t)γk−γ0]

The vector opposite to P0kc is the location of the most significant corner C0 in pose frame Lk. Note that C0 is a feature corner which is only an abstraction that the identification of the feature shall be achieved either in the histogram correlation or the LiDAR image comparison. When the LiDAR image comparison of the entire space is not computationally efficient, then the histogram correlation may not be accurate enough. An efficient way to distinguish a feature corner may be done while updating. In the linear quadratic estimation, we are able to estimate the IMR navigation velocity L˙˜k from Equation (23). Based on the estimation, we can predict the location of the C0 in pose frame Lk through computing

(34) P˜0kc=Nkt−Nk−1t+T·L˙˜k

The feature corner C0 shall be in the neiborhood of −(Lkt+P˜0kc) in LiDAR pose frame Lk. We only check the vicinity of −(Lkt+P˜0kc) of the histogram Hkx and Hky to locate the wall corners Pkc,i, which must satisfy the following condition.

(35) |P˜0kc−P0kc,i|<rs

$r_{s}$ denotes the radius of tolerance of search. The one which uses histogram search is more efficient, thus can be processed before the LiDAR image comparison. It could sometimes happen that the feature corner C0 is missing in the frame of Lk since it may be blocked by moving objects such as humans. We then have to locate the feature corner CK in LiDAR pose frame Lk−1 as follows.

(36) P0kc≈−Pkk−1c

On the way that IMR navigates, the MSC can change from one to the other and sometimes it can even loop back to an early used MSC. The update scheme for translation can then be written as follows.

(37) Nk=[Pk−1kc+(Lkt−Lk−1t)γk−γ0]=[∑i=1i=kPi−1ic+(Lkt−L0t)γk−γ0]

Pi−1ic is a zero vector when the MSC is not changed from pose frame i−1 to i.

The LiDAR images are comparing to the LiDAR mesh mk and mk−1 projection on their individual XY plane and finds the maximum correlation between the mesh image, which is the same as what has been done in conventional image processing. The translation found between Nkt and Nk−1t can be thought as P0kc. The image of LiDAR mesh can be first projected to the XY plane and compared to one another after the rotation update. The image correlation between two projected pixel images can yield the translation between Nkt and Nk−1t in a precise way, however, this is a time-consuming process and is only acquired when necessary.

## 3. Floor Management

There could be only one LiDAR pose frame L0 for the entire floor to which all LiDAR mesh data will be mapped back. However, the maximum number of quads is a finite number according to the azimuth and number of rings. In case of a complicated space such as the office building, due to several rooms and partitions, it becomes impossible to map all walls of different rooms into one simply connected mesh. Multiple connected meshes are necessary to contain the complete information of different rooms. As for floor management, we would need different room mapping even if there is only one LiDAR pose frame L0, called the base pose frame.

As stated in Section 2.2, three proposed conditions are applied as the filters to form the LiDAR mesh, which include (a) the cluster condition that is used to remove the unwanted information from the mirror reflection and those from the walls outside of the transparent windows, (b) the vertical condition to remove the passer-by and the static furniture data, such as sofa and tables, and (c) the aspect ratio condition to remove the stairstep surfaces which are not continuous walls. The adjustment of parameters used in the three filtering conditions can improve the adaptation to office buildings of different kinds. The preferences stated in Section 2.3 are that the initial location we find to turn on the IMR is better to include the rectangular room condition and the no mirror condition for increasing the estimation accuracy of the minimum area method. Since the base pose frame L0 is the basis of the IMR exploration, it determines the accuracy of the Lidar mesh map for a room. IMR navigates in the environment with assumed conditions that (1) the rotation between LiDAR pose frames is continuous, i.e., it will not fumble, and (2) there is at least one most significant corner that we can use to form the LiDAR pose frame and track the IMR navigation. These assumptions and presumptions about the environment are not limiting the use of e-SLAM, while on the other hand, they are the dimensions to improve the applicability of the e-SLAM.

### 3.1. Single Room Mapping

After a series of point cloud conversions of multiple points of different ${\hat{r}}_{n.a}$’s and same $\Psi_{n}\;,\;\alpha_{a}$ are mapped into base pose frame L0. Λk0 is the LiDAR data point obtained in pose frame Lk and converted into the LiDAR data point in pose frame L0. Θk0 is a homogenous transformation from pose frame Lk to the pose frame L0, which can be written as follows.

(38) Θk0=[RkNktO1]Λk0=Γ−1{Θk0P^k}

The total LiDAR image could be computed from the average of all range data ${\hat{r}}_{n.a}$ on the same $\Psi_{n}$ and $\alpha_{a}$ as follows.

(39) r^0n.a=∑i=0kr^in.aNn,a

$N_{n,a}$ denotes the number of non-zero range data r^in.a from all pose frame Li. In order to filter away the LiDAR data which are from the moving objects such as passer-by, we extract the LiDAR mesh data representing only the walls via the filtering conditions including the vertical condition and the aspect ratio condition as stated in Section 2.2. The wall mesh of the same area is repeatedly obtained from different LiDAR pose frames as the IMR navigates, and thus, the wall smoothing is achieved when the moving objects are far away.

### 3.2. Room/Corridor Segmentation

**Figure 10:**
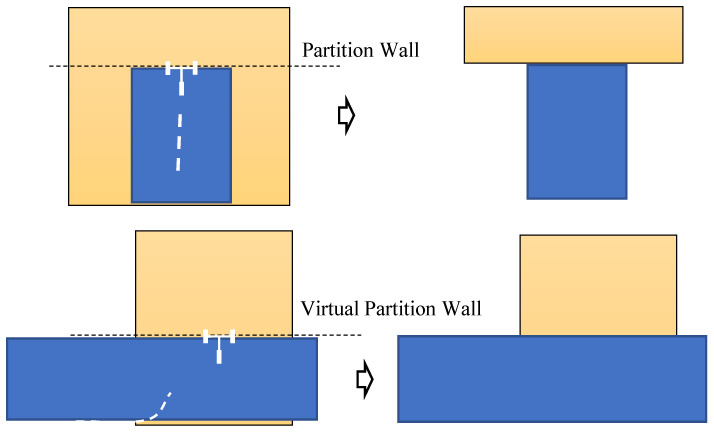
Determination of partitioned wall between rooms.

The range data of a single ray will be used to represent two walls, as stated in the previous section. There is a need to separate rooms that are partitioned by walls. We also need to label each room with an individual room index providing that the room adjacency matrix is documented. It may be convenient to setup the IMR travel distance for determining the bounding box Bk, and after determining the bounding box, the room size is fixed. It is then using the information of the IMR crossing the boundary of the box Bk to determine the wall partitioning the rooms is shown in Figure 10. When the corridors are partitioned into two rooms, there is no actual wall, however, a virtual wall is partitioning the space. As shown in Figure 10, when the IMR is leaving an initial room (blue box) and going forward for further exploration, the data on the left and right sides of the room are the LiDAR noise came from either the mirror reflection or the transparent windows. The partition wall that provides a border to the new room can filter away those noise and initialize the LiDAR mesh for a new room reached, i.e., a corridor (yellow box) in this case. As shown in Figure 10, when the IMR is turning left to a new room (yellow box) as an example, the previous data obtained from the LiDAR visible region shall be granted to the early room (blue box). A virtual partition wall (not existing) yields an open space in the mesh data storage for the new room to form its new room boundary.

## 4. Experiment and Comparison

**Figure 11:**
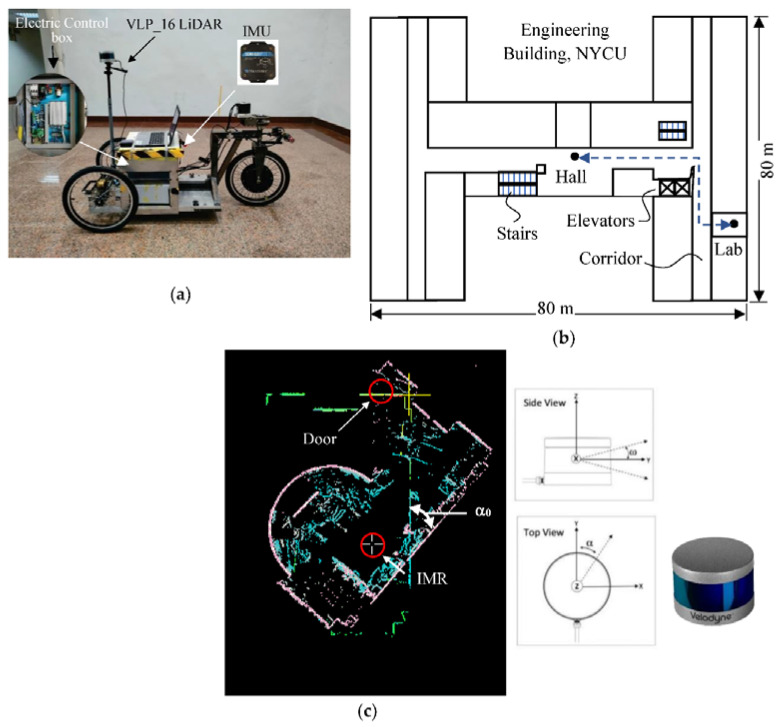
(**a**) The IMR, (**b**) top view of the floor, (**c**) calibration of LiDAR alignment.

A tadpole model of a tripod IMR is used in the experimental study. A traction in-wheel motor is applied on a single rear wheel which is integrated with an electric steering system on the top of the steering column, and two passive wheels are installed on the front wheel with 20 × 1.65 tube tire type. A VLP_16 LiDAR with 16 rings made by Velodyne Inc. is positioned onto the center of the front axle with a total height of 1.65 m from the ground as shown in Figure 11a. The tripod IMR has a turning radius of 1.2 m and is driven by people during the tests. There was an electrical control box that enclosed a 24-V drive with EtherCAT communication protocol for steering, a 48-V drive for traction controls, a 45-Ah 48-V Li-ion battery for traction system, and an IMU (Microstrain^®^ 3DM-GX1) [71]. The IMU integrating three angular rate gyros with three orthogonal DC accelerometers, which were used for the later verification purpose but not for the localization purpose. The test environment is the Engineering Building V in the Kwang-Fu Campus of the National Yang-Ming Chiao-Tung University. It is an I-shape building with two wings and a connection section. The experiment was performed on the fifth floor with a floor area of around 5000 $m^{2}$ where the top view is shown in Figure 11b. The wall material is made from concrete and white fiber cement layered onto the wall surface with a solar reflectance of 0.40. There are four tests moving repeatedly from the hall to the lab. A simple text file in pts format is used to store point data from LiDAR scanners. The first line gives the number of points to follow. Each subsequent line has 7 values, the first three are the (x, y, z) coordinates of the point, the fourth is an “intensity” value, and the last three are the (r, g, b) color estimates. The intensity value is an estimate of the fraction of incident radiation reflected by the surface at that point where 0 indicates no return while 255 is a strong return. In the lab, there is a dome screen made of wood, which occupied one quarter of the lab space. There is also a mirror on the wall adjacent to the door which can produce fake feature data by reflecting the laser light. As shown in Figure 11c, the IMR is in its upfront position in the room toward the door. The angular difference between the LiDAR data and the LiDAR mesh is the initial azimuth angle $\alpha_{0}$. After the initial azimuth angle is obtained from the proposed method in Equation (15), one can reinstall the LiDAR by manually rotating the LiDAR fixture on the IMR to align the zero-azimuth angle to the upfront direction. There will still be some inaccuracy left behind needed to be verified through the experiment.

There are two tours from the lab to the hall and from the hall to the lab. Each of the tours has been performed twice. The intent of the course of the experiment is to prove the generality of the proposed method as follows.

(1) In the course of the experiment, IMR went from a door-closed room to the corridor after the door is opened. The challenges include the complication of LiDAR mesh formation from the closed area to an open area, and also the floor management.
(2) The IMR navigates from a room to the corridor and to the elevator space where many passengers (moving objects) are presented, and then, to the lobby which is an open space. The corridor navigation is a challenging part, which has very weak MSC information when the walls do not intersect.
(3) The IMR makes two 90 degree turns, one right and one left, between two complete stops. The displacement and orientation estimation of the proposed method can be fully tested.

During the tour from the lab to the hall, there are four most significant corners (MSC) found in the lab, more than 10 MSC’s found on the corridor and many other MSC’s found during the entire IMR navigation. There is one person driving the IMR manually by its handle bar which includes the throttle and brake to control the IMR. The person who controls the handle bar is at his lowest position as possible to avoid blocking the LiDAR rays from shooting on the high side of the walls.

**Figure 12:**
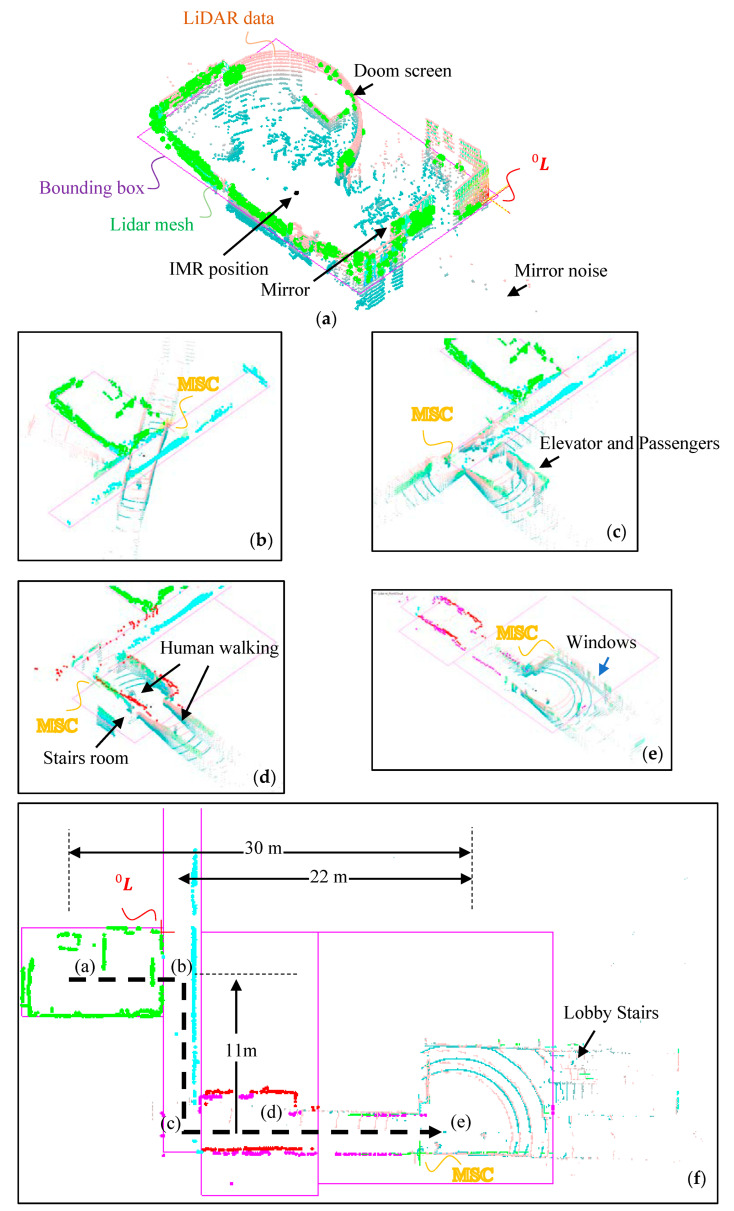
IMR navigation from (**a**) Lab and proceeds to (**b**) Corridor, (**c**) T-junction, (**d**) Elevator room, (**e**) the lobby, (**f**) e-SLAM map.

We chose only point clouds from positive ring angles $\Psi_{n}$ to form the LiDAR mesh in the experiment. We assumed that the high portion of the wall poses more wall corner information than the lower portion does. Thus, in VLP_16, there are $\Psi_{n}=1,3,\;5,\;7,9,\;11,\;13,\;15$ degrees that can be utilized to form the mesh. In different experiments, we have collected 550 to 750 LiDAR data frames from four different IMR navigations within 80 to 120 s. The frame rate is around 5 data frames per second that we recorded. The total distance accumulated during the navigation is about 45 m. The average speed of the IMR is 2 kph (km/hour) which is a medium speed in the indoor mobile robot application. During the navigation, there are two T-junctions to turn and the IMR will stop when there are people walking by. The highest speed of the navigation was 4 kph during the course. In Figure 12, we demonstrate the tour from the lab to the hall. There is a dome screen which is nearly one quarter area of the lab, which also provides a counterexample of vertical walls in this room. There is also a mirror on the side of the entrance door, thus providing a LiDAR error source. The base pose frame L0 is chosen to be the first found MSC when IMR starts the navigation, which can also program to other locations in order to have a better resolution of the entire space with a different room. In Figure 12a, we can find two images, for which the real time LiDAR data and LiDAR mesh were calculated. They almost coincide with each other just because when the IMR started navigation, the LiDAR azimuth angle $\alpha_{0}$ is pre-aligned to front. The alignment of LiDAR azimuth angle $\alpha_{0}$ to the front of the IMR is not always true in applications of multiple LiDAR systems. Indeed, it is very difficult to align the LiDAR azimuth angle $\alpha_{0}$ precisely to front and the misalignment can be calculated during the first minimum bounding box test. The angle between the bounding box and the IMR is the misalignment angle. In Figure 12b, we demonstrate the circular motion of the IMR. During the circular motion, the IMR merely moved and the LiDAR scanner senses only the rotation. In Figure 12c, when the IMR reaches the T-junction between the hall way and the corridor, the IMR rotates again. Between Figure 12b to Figure 12c, there are many number of MSC jumps since the corridor does not have the significant MSC which is seen by the LiDAR scanner. Thus, MSC may jump to the frame of the door and also to the bulletin box on the walls which indeed needs the LiDAR mesh image comparison between the room and the current rotated LiDAR mesh data stated early in the final paragraph in Section 2.8 “Translation Update Scheme based on Most Significant Corners (MSC) Transfer”. In our software, we will send a beep sound out from the controller of the IMR which signals the difficult situation and possibly error translation updated. In practice, there will be many other sensors such as GPD and IMU in addition to the LiDAR scanner helping advise on the correction translation. However, in this experiment, we purposefully do not acquire the information from the other sensors to fully understand the reliability of the e-SLAM method for the localization of IMR. In Figure 12d, when the IMR is moving to the elevator room, there are passengers waiting for the elevator, while one of the passengers was entering the elevator, thus, the elevator door was opening and closing. In Figure 12e, IMR reached the hall and before that there was a person walking through the hallway and encountered the IMR. It can be seen that the floor management is performed to separate the entire course into 4 rooms where one is adjacent to the other. At the top view of the map produced from the e-SLAM as shown in Figure 12f, there are several things found which shall be discussed.

(1) The LiDAR azimuth angle $\alpha_{0}$ is not zero. From the top view of the LiDAR mesh image, it is found that the final LiDAR data are not perfectly aligned with the map.
(2) The room near to the base pose frame L0 gets better resolution. It can be observed from Figure 12f that the Lab with more green dots representing the LiDAR quads and the lobby received fewer dots. It is because the resolution of the LiDAR quad is limited by the distance from its base pose frame L0. We can have a quad with one meter height because the distance was 30 m away from the base pose frame L0 and the ring angle resolution is 2 degrees.
(3) There is noise involved into the room bounding box which can be caused by the reflecting image from the shining surface such as windows of the bulletin.
(4) The maps of room (d) and room (e) with different colors overlapped each other. It is also observed from all adjacent rooms. The overlap region can allow us to tell the dislocation of the translation update scheme.
(5) The room segmentation is determined by the IMR travel distance and the bounding box is applicable when the rectangular room is detected. However, it failed to box precisely the space when it was on the corridor and the hallway. This can cause an even bigger problem when IMR travels on a long corridor.

**Figure 13:**
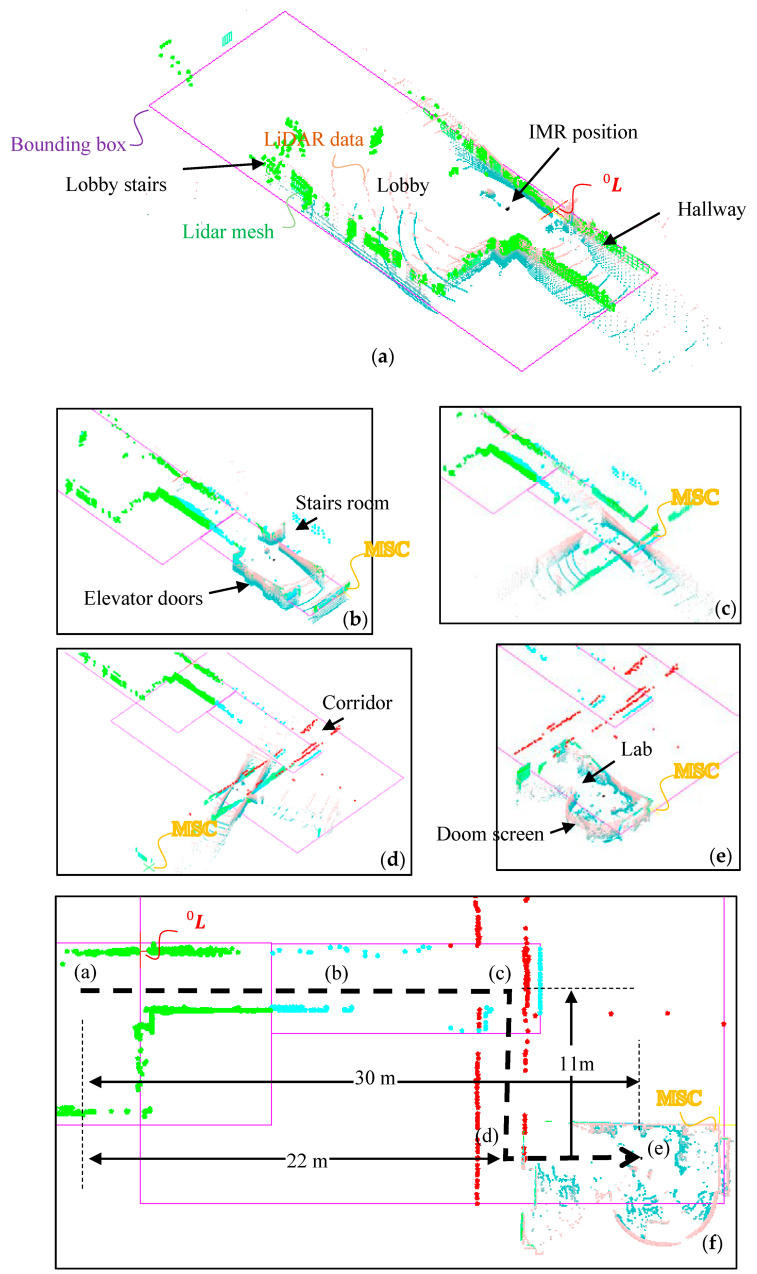
IMR navigation from (**a**) the lobby and proceeds to (**b**) Hallway, (**c**) Corner, (**d**) Corridor, (**e**) the lab on, (**f**) e-SLAM map.

The other test is conducted by moving the IMR back to the lab. In Figure 13a, we see that the LiDAR data on the vicinity of the hallway had ring arcs in different wave spacings. The denser ring arcs are the ceiling. The sparser ring arcs are the hallway. It looked like the hallway had an inclined floor surface, which is actually flat and horizontal to the ceiling, since the wave spacing changes of both floor and the ceiling are the same. In Figure 13a, the rectangular bounding box can still be found in even a very complicated space. The base pose frame L0 is also chosen to be the first found MSC when IMR starts the navigation. However, this time, the MSC formed is a virtual corner which is the intersection of non-touching walls. In Figure 13b, the IMR travels through the hallway and sees the stair room, which is then moving to the T-junction between the hallway and the corridor as shown in Figure 13c. In Figure 13d, the IMR reaches the door of the Lab and IMR continues to go further into the Lab. Figure 13f shows the top view of the map produced from the e-SLAM, it can be observed that the room index is not successfully provided to the Lab because of the low resolution of the Lab when it is far away from the base pose frame L0. It is also observed that the third room including the hallway, the corridor and the Lab cannot be separated from the hallway because there is no good separation line found when the IMR moved from hallway to the corridor. The T-junction occupied by the hallway room blocked the corridor to be formed as a unit of room. 

**Figure 14:**
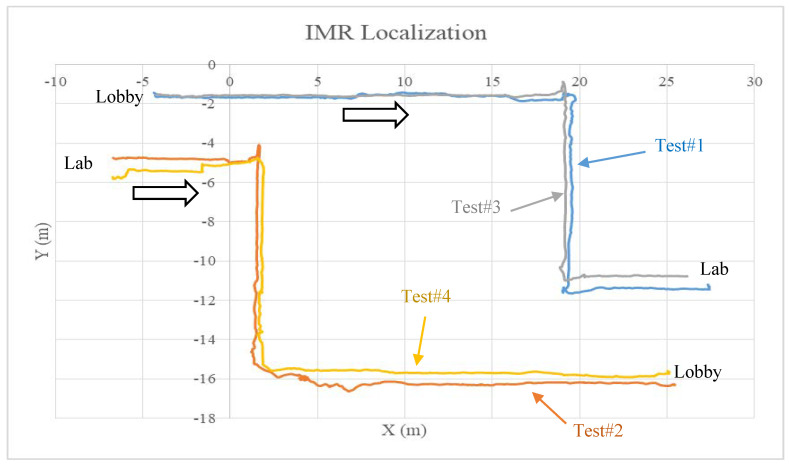
Active localization of IMR based on e-SLAM.

The other two tests are repeated from the previous two tests, which are used to compare the rotation and translation reading of the IMR based on the active localization based on the e-SLAM method. Each of the tests are performed with different navigation speeds and waiting times when encountering moving persons, with different number of data frames from 550 in Test#3 to 750 in Test#1. The results are compared in Figure 14, the x- distance between the lobby anchor points to the Lab anchor point is about 30 m, the lobby anchor point to the T-junction is about 22 m, and the T-junction to the position in front of the door is about 11 m. Each time, the IMR trajectory may be different by 1 m. The direction of IMR was rotated at 180 degrees every time before the next test started. Thus, their coordinate systems are different in x-direction, i.e., the x-direction of the tests from lobby to Lab is opposite to that of the tests from Lab to lobby. They matched in dimensions of the space within meter accuracy. The amount of error may due to the causes in the control and analysis.

(1) The trajectory difference: There have been real situations in different IMR navigation tests including passer-by, elevator passengers, and environmental conditions such as door open/close. These situations caused the non-holonomic robot control system to detour from the predefined runway, which has been recorded in video files allowing replay to verify. So far, it contributes 50% of the error based on the video replay data.
(2) The starting and ending position difference: The starting positions are marked before each test, however, precisely arriving at the same mark as the ending position is difficult for the IMR due to the position control being done manually using electrical throttle and brakes as stated before. The differences contribute 30% of the error so far.
(3) The e-SLAM localization error: There still were some errors found from the simulation analysis in the localization process. These errors will show the LiDAR meshes slightly shaking on the screen in the simulation streaming. The computation will contribute 20% of the error based on observing the simulation streaming.

**Figure 15:**
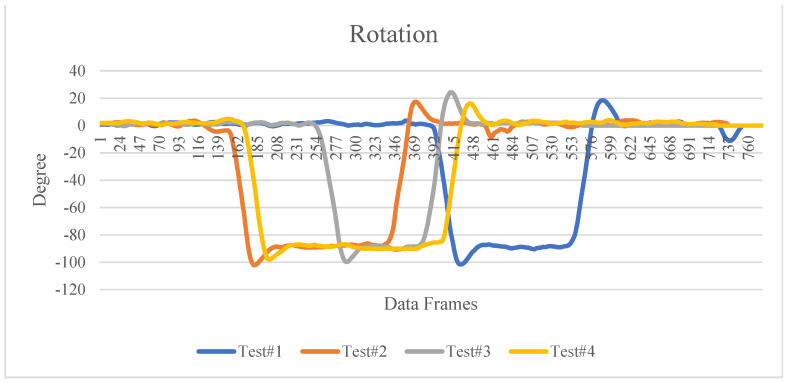
IMR rotation detected by e-SLAM.

The error of Test#4 is largest among all four tests, which may be caused by the e-SLAM localization error. Thus, we may conclude that the deviation of the active localization of e-SLAM is within 50 cm. This error may need the compensation from other sensors such as IMU or high precision GPS to compensate. It may also acquire the precise calibration of the locations from the image recognition of fiducial marks. As for the rotation based on the active localization, all four tests are done by first going along a straight line with no rotation, then turn to the right by 90 degrees, then travel on the corridor in a straight line, turn to the left by 90 degrees, and then going straight. Since they have different number of data frames, they cannot be compared at the same time count. The result in Figure 15 shows that there is always an overshoot during rotation which was actually happening during the IMR navigation. The maximum rotation overshoot is around 20 degrees. Comparing to the actual data frames played as a movie, we can observe only less than 5 degrees of error for the rotational localization, thus, we can conclude the error of rotation is within 5 degrees. The error may be compensated by the gyroscope in the high precision IMU. The initial azimuth angle $\alpha_{0}$ does not affect the relative rotation information between LiDAR pose frames which yields the rotation of the IMR. Under the condition mentioned previously that the LiDAR azimuth angle $\alpha_{0}$ is non-zero, the rotation results as shown in Figure 14 have two steady states on precisely 0 and 90 degrees which are not affected by the LiDAR azimuth angle $\alpha_{0}$. Hence, further calibration of the initial LiDAR azimuth angle $\alpha_{0}$ is unnecessary, and it can reduce the maintenance work.

**Figure 16:**
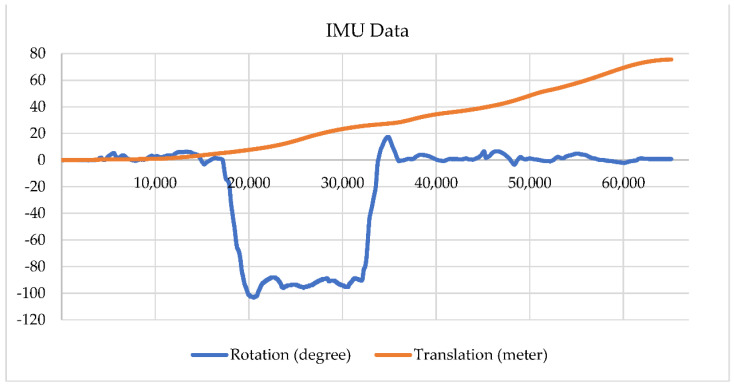
IMU data recorded on the tour of Test#4.

In order to show the significance of the proposed system, we also show the comparative data obtained from the IMU shown in Figure 16. The rate for Euler angles is 100 Hz and for accelerometer data is 350 Hz from the IMU. The horizontal axis is the data count instead of the actual time. Figure 15 and Figure 16 are from different sampling rates collected from different routines, thus, they had different data counts. The IMU data were recorded on the tour of Test#4. It is found that the rotation angle (blue line) in Figure 16 is coarsely comparable to the Test#4 result (yellow line) shown in Figure 15, however, IMU rotation data are noisier than those found from the e-SLAM result on straight line sections. The overall travel distance on the tour of Test#4 shall be 41 to 42 m according to Figure 12, which is different from the translation of 75 m calculated from the accelerometer result shown in Figure 16. The IMU travel distance error may be due to the calibration of the accelerometer. As a result, the e-SLAM method proposed in this paper is suitable for position accuracy as well as rotation stability. The e-SLAM is not an integral-based sensor, which is free from accumulation error.

**Figure 17:**
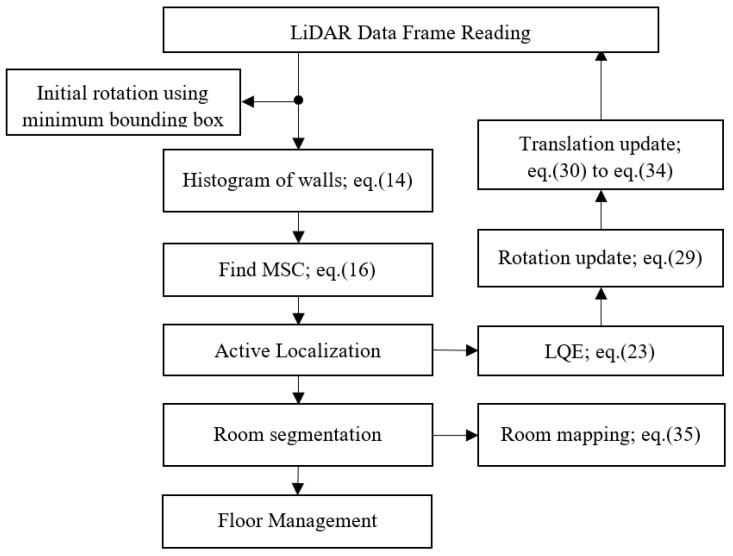
Flowchart of e-SLAM.

In summary, the e-SLAM includes the procedures of (1) finding the initial rotation using the minimum bounding box method, (2) finding the most significant corner from the walls vertical to the floor based on the histogram analysis, (3) choosing the base LiDAR pose frame and doing the inverse transformation to map all LiDAR data back to the room map, (4) performing the active localization based on the rotation and translation update scheme, (5) utilizing the least quadratic estimation (LQE) method to perform the localization estimation, (6) utilizing the LiDAR data XY projection image to assist the translation update, (7) performing the room segmentation, and (8) performing the floor management. The flow chart of the e-SLAM processing is shown in Figure 17.

## 5. Conclusions

The method of exploration-based SLAM (e-SLAM) for the indoor mobile robot using LiDAR is introduced in this paper. The e-SLAM method with the mapping using LiDAR mesh of vertical walls can trim the static furniture and the moving object from the same space of the IMR through the proposed conditions in Section 2.2 for the LiDAR mesh formation. With the help from LQE estimation on the translation and rotation, LiDAR can be the only sensor used for the active localization. The computation time is 90 s to process the longest PTS file with 750 LiDAR data frames, which recorded 120 s of the IMR navigation, using a computer with a Windows10 operating system and an Intel(R) Core (TM) i5-8500 CPU @ 3.00 GHz 3.00 GHz CPU and 4 GB memory. The software was running in a single thread executable code. The e-SLAM can achieve the real-time processing. The precision of e-SLAM is 50 cm in translation and 5 degrees in rotation. When the most significant corner in starting position of the IMR is made as the base pose frame, the maximum distance for the 16-ring LiDAR can go as far as 35 m away to persist good active localization. When the space requires more than 35 m, multiple base pose frames can be used to increase the resolution during the XY projection image comparison. The experiment was conducted during the office hour of NYCU engineering building when many moving objects such as humans and elevator doors were into the LiDAR scanning. The reliability of the e-SLAM method is preliminarily proven. There are still problems left unsolved to the future, which include the method to handle multiple base pose frame L0 and a better room segmentation method to handle the long corridors. Nevertheless, there is a need to improve the translation update accuracy.

## Data Availability

Data sharing is not applicable.

## Nomenclature

**Symbol**	**Abbreviation**
Ψ	Angle formed between rings from the LiDAR center
α	Azimuth angle
$\Lambda_{n.a}$	LiDAR data point at *n*-th ring by *a*-th ray
Γ	LiDAR transformation matrix
${\hat{P}}_{n,a}$	LiDAR data point in the rectangular coordinate system
Lk	LiDAR point at pose frame *k*
$Q_{n,a}$	Quad in mesh formed at *n*-th ring by *a*-th ray
${\hat{n}}_{Q,n,a}$	Normal vector at quad $Q_{n,a}$
mk	Mesh at pose frame *k*
Bk	Boundary box at mesh mk
γ	Angle for rotation at XY plane for LiDAR image projection
$W_{x},W_{y}$	X walls, Y walls
Ck	Most significant corner (MSC) at pose frame *k*
IMR	Indoor Mobile Robot
Nkt,Nkγ	Translation of IMR, Rotation of IMR at pose frame *k*
